# Dynamics of natural populations of the dertitivorous mudsnail *Potamopyrgus antipodarum* (Gray) (Hydrobiidae) in two interconnected Lakes differing in trophic state

**DOI:** 10.1186/2193-1801-3-736

**Published:** 2014-12-15

**Authors:** Jaap Dorgelo, Harm G van der Geest, Ellard R Hunting

**Affiliations:** Institute for Biodiversity and Ecosystem Dynamics, Aquatic Ecology and Ecotoxicology, University of Amsterdam, PO Box 94248, NL-1090 GE Amsterdam, The Netherlands; Department of Conservation Biology, Institute of Environmental Sciences (CML), Leiden University, Einsteinweg 2, NL-2333 CC Leiden, The Netherlands

**Keywords:** *Potamopyrgus antipodarum*, Detritus, Trophic states of lakes, Growth, Reproduction, Resource allocation

## Abstract

Here we investigate the allocation of resources between growth and reproduction by surveying the dynamics of natural populations of the aquatic detritivorous mudsnail *Potamopyrgus antipodarum* from two interconnected lakes that differ in trophic state. The size distributions and reproductive output of the snail populations was analyzed monthly by field surveys spanning 3 years, and in a controlled microcosm experiment to evaluate the reproductive potential under laboratory conditions. Snails in the meso-oligotrophic lake showed reduced growth and a smaller size compared to snails in the eutrophic lake. However, the numbers of eggs and nearly-neonates per adult snail did not differ significantly between the two populations. It is speculated that *P. antipodarum* populations living under meso-oligotrophic conditions may consistently invest more internal energy in reproduction at the expense of growth and that food quantity may be an important driver for macro-invertebrate resource allocation in detrital food webs.

**Electronic supplementary material:**

The online version of this article (doi:10.1186/2193-1801-3-736) contains supplementary material, which is available to authorized users.

## Introduction

The New Zealand freshwater mudsnail *Potamopyrgus antipodarum* gained increased interest in the last decades since it has successfully invaded a large number of ecosystems across the world, including Europe, Australia and North America (Frömming^1956^; Ponder^1988^; Hall et al.^2006^; Vinson and Baker^2008^), and it is increasingly used as a model organism for ecotoxicological studies (e.g. Duft et al.^2007^; Gust et al.^2011^; Coulaud et al.^2013^). The mudsnail *P. antipodarum* is a relatively small (≤ 6 mm) dioecious snail that feeds on detritus by surface scraping (Haynes and Taylor^1984^). Aquatic invertebrates that feed on dead organic matter (detritus) often live in environments with limited and variable quantities and qualities of food, and consumer growth and reproduction is well known to be influenced by the chemical composition or quality of their food (e.g. Kampfraath et al.^2012^; Lau et al.^2013^). Detritus has a low nutritional value, which is compensated by ingesting large amounts of material that passes rapidly through their gut and is only partly digested (e.g. Lopez and Levinton^1987^; Wotton^1994^). Large quantities of detritus are thus required to sustain populations of detritivores. Indeed, populations of e.g. deposit-feeders are often food-limited (Gee^1988^; Forbes and Lopez^1990^; Richardson^1991^) and food enrichment has been shown to increase growth (Dorgelo^1991^) and fecundity (e.g. Osenberg^1989^; Dorgelo and Leonards^2001^; Richardson^1991^; Lemke et al.^2007^). This suggests that, although various life history strategies have been observed for aquatic invertebrates (cf. Thompson^1982^; Rollo and Hawryluk^1988^; Dorgelo^1993^; Chase^1999^), lakes with high(er) primary productivity and detritus inputs more likely stimulate growth and reproduction of aquatic invertebrates. Recent laboratory studies indeed confirmed that the reproductive output in populations of the mudsnail *P. antipodarum* can be negatively affected by food limitation (Neiman et al.^2013^), and that growth and fecundity are negatively affected by food quality (Tibbets et al.^2010^; Neiman et al.^2013^). However, long-term studies on natural populations are lacking.

Here we report on a three year survey of two populations of *P. antipodarum* in two interconnected, yet contrasting freshwater lakes: a meso-oligotrophic lake and an eutrophic lake. We focused on the relative distribution of internal energy between somatic growth and reproduction by sampling the snail populations over a three year period and analyzing brood pouch content and shell size classes. The reproductive potential of both populations was tested in an additional laboratory microcosm experiment.

## Materials and methods

### Study site

**Figure 1:**
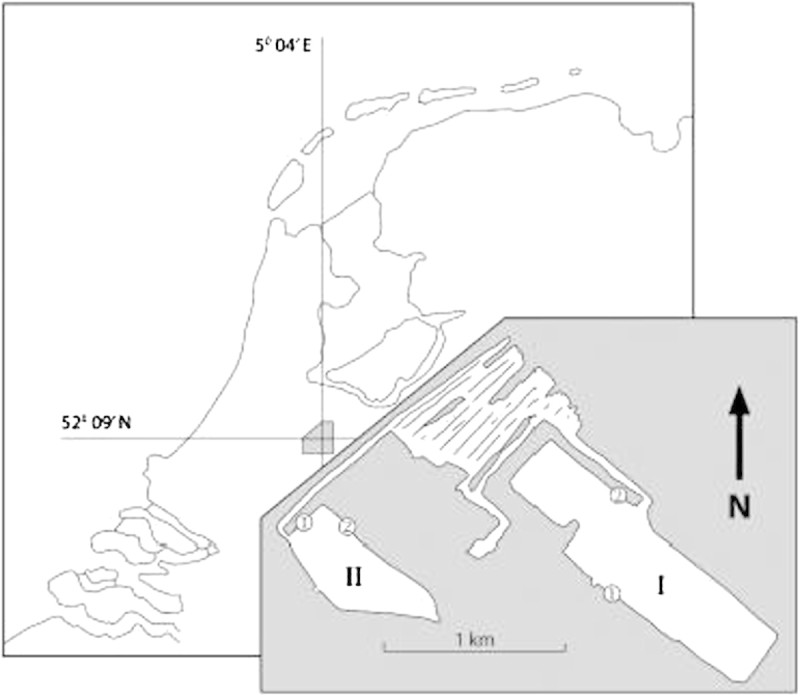
**Geographic location of the Maarsseveen Lake system in The Netherlands (from Swain et al** ***.***^1987^**).** I. Meso-oligotrophic lake I; II. Eutrophic lake II. 1. Locations of population analysis; 2. locations of snails used for brood pouch analysis.

**Figure 2:**
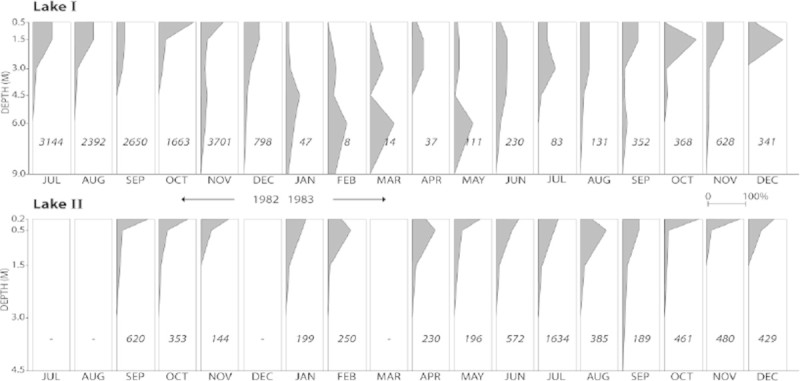
**Relative distribution of** ***P. antipodarum*** **over depth in meso-oligotrophic lake I and eutrophic lake II.** Depth in m. Snail abundances (grey areas) are presented as the percentage of the total number collected monthly (indicated by the numbers) to allow comparison of the relative distribution between meso-oligotrophic lake I and eutrophic lake II, in which the grey area accounts for 100% of the sampled time frame for each lake.

The samples for the present snail population study were collected at sandy bottom locations without macrophytes of the meso-oligotrophic Lake Maarsseveen I and the eutrophic Lake Maarsseveen II. The values of the maximum primary phytoplankton productivity were 500 and 8,125 mg C.m-2. day-1 in the meso-oligotrophic and eutrophic Lake, respectively (Table 1). The two man-made lakes, resulting from sand extraction in the 1960s, are situated close to each other (approx. 1 km distance) in the center of the Netherlands to the northwest of the city of Utrecht (Figure 1). The lakes are interconnected by water channels, but the meso-oligotrophic lake is mainly supplied by upwelling groundwater from the higher region in the east while the eutrophic lake is mainly supplied by water from the nutrient-rich river Vecht. The mudsnail *P. antipodarum* invaded these areas in Europe around the 1970s. The close vicinity of both lakes provide the unique opportunity to evaluate the effect of trophic state on *P. antipodarum* population dynamics without confounding effects of potentially widely differing parameters such as physico-chemical conditions (Neuparth et al.^2002^; Schreiber et al.^1998^), parasites (Krist and Lively^1998^) and predation pressure (Whitehead^1935^; Boettger^1951^; Vinson and Baker^2008^) that are known to strongly influence on *P. antipodarum* population dynamics. The presence of sandy substratum was limited in lake II, since most of the littoral consisted of mud covered with (partly decayed) tree leaves (aquatic macrophytes were very rare in this lake) and woody debris, a habitat in which snails did not occur. At the sampling station this type of substratum was also found from approximately 1.5 m depth onward. In the meso-oligotrophic lake, the sandy region was far more extended over depth. Here, the natural detrital food of *P. antipodarum* mainly originates from phytoplankton blooms and autumnal pulses of leaves from surrounding trees, supplemented with material from macrophyte stands. A detailed description of the seasonal periodicity of phytoplankton (very pronounced in diatom species) in the meso-oligotrophic lake was reported by Dorgelo et al. (1981). No such analysis of the eutrophic lake is available, but here, autumn leaves are the low nutritional bulk source of detritus (Vos et al.^2000^). The nutritional importance of diatoms in the diet of deposit feeders has often been recognized (references in Dorgelo and Leonards^2001^). Both lakes, though most pronounced in the eutrophic lake, showed summer maxima of the cyanobacterium *Mycrosystis aeruginosa*, but no data are available of possible toxic effects of e.g. *Planktothrix agardhii* (see Lance et al.^2007^) on the macrofauna in these lakes. Both lakes have minimal fluctuations in water level and show summer stratification (for further lake characteristics, see Table 1). Summer oxygen profiles were measured (Figure 2) by means of a Y.S.I. (Yellow Springs, Ohio, U.S.A.) dissolved oxygen probe.

**Table 1:** **Selected characteristics of the two lakes**

	Meso-oligotrophic lake	Eutrophic lake
Year created	1960	1967
Surface area (ha)	70	20
Max. depth (m)	30	25
Range 1% light depth (m)	7–11	3–7.5
Range P.PO_4_ (μg.1^-1^)	0–2	8–304
Range total P (μg.1^-1^)	3.5–17	151–365
Range N.NO_3_ (μg.1^-1^)	130–350	30–1,400
Max. primary phytoplankton prod. (mg C.m^-2^.day^-1^)	500	8,125
Ca^2+^(mg.1^-1^)	61	75
Conductivity (μS.cm^-2^)	355	550
pH*	8.2	7.9

Nutrient concentrations at 0.5 m depth. For references see Dorgelo and Gorter (1984).

*taken from Swain et al. (1987).

### Population size distribution and reproduction

Prior to the survey, the depth distribution of *P. antipodarum* was established during 18 successive months by means of a Petersen grab which proved equally efficient as the suction sampler used later (Dorgelo and Hengst^1986^). Population size distribution and reproduction were subsequently quantitatively sampled monthly during 1984–1986 at permanent stations by means of two modifications of a hydraulic lift sampler. One version was used in shallow water (suction diameter 160 mm), while the other was only appropriate in water deeper than 1.5 m (suction diameter 125 mm; Dorgelo and Hengst^1986^). At each visit (January of the second year was excluded due to ice cover), 20 samples between 0.5 and 4 m depth were taken in the meso-oligotrophic lake, and 10 samples between 0.2 and 1.5 m depth in the eutrophic lake because most individuals were restricted to the shallow depths in the eutrophic lake. During summer stratification these depth ranges are within the oxygenated layer of the water column (Figure 2). Snails were transported alive to the laboratory and, due to the large numbers involved, stack sieved (using sieves with slit-shaped perforations) into six size classes according to the width of the shell whorl. The allometric relation between shell width and length of *P. antipodarum* populations in these lakes was studied prior to this study and is described in Dorgelo (1991). Samples were sieved into shell width ranges of 0.60–1.00 (exclusive), 1.00–1.25, 1.25–1.50, 1.50–1.75, 1.75–2.00, and ≥ 2.00 mm (only a few individuals were found with shell widths larger than 2.50 mm, and therefore all widths ≥ 2.00 mm were pooled). These sieve fractions, henceforth distinguished as 0.60, 1.00, 1.25, 1.50, 1.75 and 2.00 mm snails, were isolated by a flow of copper-free tap water pressing through the perforations and quantified. Later the snails were set free at the sampling stations in order to avoid a sampling predation effect. Provided that the data followed a normal distribution (Shapiro-Wilk test on residuals), we used a general linear model (GLM) approach to analysis of covariance (ANCOVA) to compare snail size distributions between lakes (Hammer et al.^2001^; Engqvist^2005^). The dependent variable was proportion of each size, and time (date) was the covariate. Since size distributions did not differ in slopes (ANCOVA, p = 0.18), size distributions were not dependent on the covariate ‘time’. Therefore, we could remove the interaction term ‘time’ to test for the effect of size distribution with a Tukey’s HSD post hoc test (e.g. Hunting et al.^2012^).

In both lakes, reproductive maturity was observed in a few specimens of the largest 1.50 mm snails, but was generally found for the 1.75 and 2.00 mm size class snails. Since 2.00 mm snails were less numerous, only 1.75 mm snails (25 per site) were used for pouch analysis. They were collected with a dip net, synchronously with the snails collected for the population dynamics analysis. Snails used for the analysis of population dynamics and brood pouch content were collected at different locations (Figure 1), again in order to avoid the predation effect. The distance between these locations, in a straight line, was approximately 500 and 300 m in the meso-oligotrophic and eutrophic lake, respectively. Reproductive activity of snails was examined by dissecting the snail and counting the content of the brood pouch under a binocular microscope. Embryos are located in the brood pouch which is formed by the enlarged oviduct. This content consisted of eggs and embryos (in which various developmental stages were pooled), and of nearly-neonates, ready to leave the pouch, with a very thin shell and moving, which were separately counted. Analysis of covariance (ANCOVA) using date as a covariate was used to assess differences in average number of unborn juveniles per brood pouch between sampling events, and between locations (Hammer et al.^2001^).

### Microcosms

To evaluate the reproductive potential of both populations, the dynamics of both populations was monitored in a microcosm experiment. The microcosm setup comprised two 10-L plastic aquaria (bottom surface 544 cm^2^). Each microcosm was provided with 8 L filtered lake water above 2 cm sediment originating from either the meso-oligotrophic lake or the eutrophic lake. Beforehand, the sand was sieved twice with an interval of two weeks to ensure removal of resident fauna: primarily small-sized chironomid larvae and ≤ 0.60 mm snails which prevented that snails present in the sediment contributed to the outcome of the microcosm incubation. This procedure also ensured a consistent grain size of ≤ 0.60 mm, thereby excluding confounding effects of sediment size (e.g. Holomuzki and Biggs^2007^). The aquaria were kept in water baths at 15.0 ± 1,0°C in a room where the light and dark periods followed the natural light regime, i.e. 16 h light: 8 h dark. Air was supplied through a pump and an air-diffusing stone. Filtered lake water was refreshed and biofilm was cleaned from the aquarium walls monthly. Detrital aggregate was, separated by lake origin, obtained from benthic surface samples mixed from various random locations in each lake and sieved through a 0.60 mm sieve. The retained sieved > 0.60 mm material was stirred in filtered lake water. Even the smallest snails and larvae proved to sink immediately after stirring the flocculent detritus. 15 ml of aqueous detritus suspension was added monthly to each aquarium, as well as 1 leaf of fresh lettuce (commonly used for rearing snails) which was replenished as soon as it was consumed entirely. It partly decomposed during the study resulting in increased palatability. Twenty 1.75 mm snails from each lake were placed in each aquarium. The numerical content of the brood pouch of 1.75 mm snails, taking together eggs, developmental stages and nearly neonates, amounted to 6.2 ± 1.0 (SE) and 4.9 ± 1.1 in the meso-oligotrophic lake and the eutrophic lake (N = 25), respectively, at the start of the experiment. These snails were sampled synchronously with the individuals placed in the aquaria. At each monthly sampling interval snails were sieved (very carefully in order to save the fragile (≤ 0.60 mm) freshly released young which were not retained on the sieve), and counted per size class. The experiment lasted nine months to ensure sufficient offspring and allow comparison of how the populations originating from either the meso-oligotrophic lake and the eutrophic evolved under microcosm conditions. The experiment started in March, 1987 in which the first counting was performed in April.

## Results

### Depth distribution

**Figure 3:**
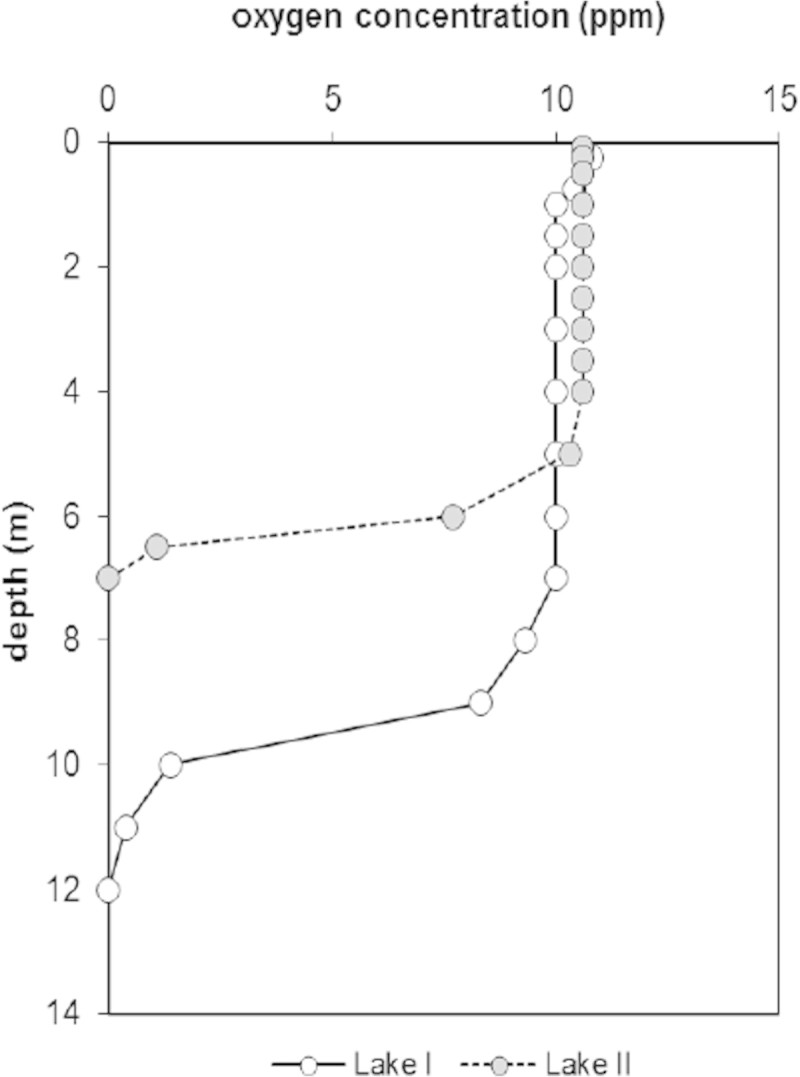
**Dissolved oxygen concentrations (ppm) during summer stratification in meso-oligotrophic lake I and eutrophic lake II.**

The depth distribution of *P. antipodarum* differed between the two lakes. In the meso-oligotrophic lake, the snails were encountered deeper and in more fluctuating numbers than in the eutrophic lake (Figure 2). Maximum numbers in the meso-oligotrophic lake occurred at a depth of 6 m before stratification in February, March and May. In the eutrophic lake, the snails were more evenly distributed over a more limited depth range. Oxygen profiles indicated that the metalimnion was located at approximately 9.5 and 6 m depth in the meso-oligotrophic and eutrophic lake, respectively (Figure 3), additionally illustrating the difference in trophic conditions between both lakes.

### Population size distribution and reproduction

**Figure 4:**
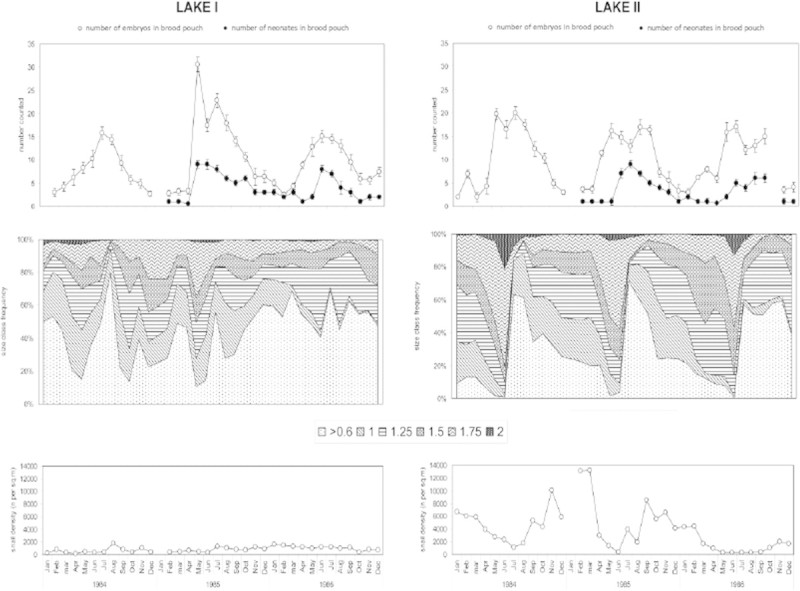
**Field data of the two populations in meso-oligotrophic lake I and eutrophic lake II.** Upper panel: brood pouch data; means ± S.E.; N_snails_ = 25; open dots denote total numbers of embryos; solid dots denote number of nearly-neonates (not counted in 1984); middle panel: monthly relative shell width (in mm) frequency distribution of snails; bottom panel: total snail densities (lake I: N = 20; lake II: N = 10). There was no significant difference in average number of eggs per brood pouch between both lakes (ANCOVA: F = 0.036, P = 0.85). There was also no significant difference in average number of neonates per brood pouch between the lakes (ANCOVA: F = 1.18 =, P = 0.28). A significantly higher contribution of larger (>1.75 mm) snails was observed in the eutrophic lake compared with the meso-oligotrophic lake (GLM-ANCOVA, Tukey-HSD post hoc, P < 0.05). Snail densities in the meso-oligotrophic lake were significantly lower than the eutrophic Lake (*t*-test; P < 0.05; bottom panel).

In the meso-oligotrophic lake, the 0.60 mm snails contributed to the population by extended presence, in an irregular, annual pattern (Figure 4, middle panel), while size-distributions followed a regular annual pattern in the eutrophic lake. The periodicity in nearly-neonate production (upper panel) was reflected by the relative abundance of the 0.60 mm snails to the population. When the 0.60 mm snails decreased, the contribution of the pre-adults increased. A significantly higher contribution of larger (>1.75 mm) snails was observed in the eutrophic lake compared with the meso-oligotrophic lake (GLM-ANCOVA, Tukey-HSD post hoc, P < 0.05). The population in the eutrophic lake thus showed a more stable structure and more 2.0 mm snails than in the meso-oligotrophic lake. In 1984 and 1985, minimum snail density occurred in the eutrophic lake in early summer, whereas this minimum continued to the end of the year in 1986 (Figure 4, bottom panel). Snail densities in the meso-oligotrophic lake were always low compared to the eutrophic lake and consequently the yearly averages in the two lakes also differed significantly (*t*-test; P < 0.05), being highest in the eutrophic lake (Figure 4, bottom panel).

In both lakes, the numbers of developmental stages (from egg to nearly-neonate) as well as the number of nearly-neonates in the brood pouch of individual adult snails were characterized by summer maxima, and some yearly variation from May to August in lake I and from May to September in the eutrophic lake (Figure 4, upper panel). During the rest of the year reproductive activity continued at a reduced rate, in correspondence with the data obtained by Dussart (1977) and Schreiber et al. (1998). During our survey, there was no significant difference in average number of eggs per brood pouch between both lakes (ANCOVA: F = 0.036, P = 0.85), and there was also no significant difference in average number of neonates per brood pouch between the lakes (ANCOVA: F = 1.18 =, P = 0.28).

### Microcosms

**Figure 5:**
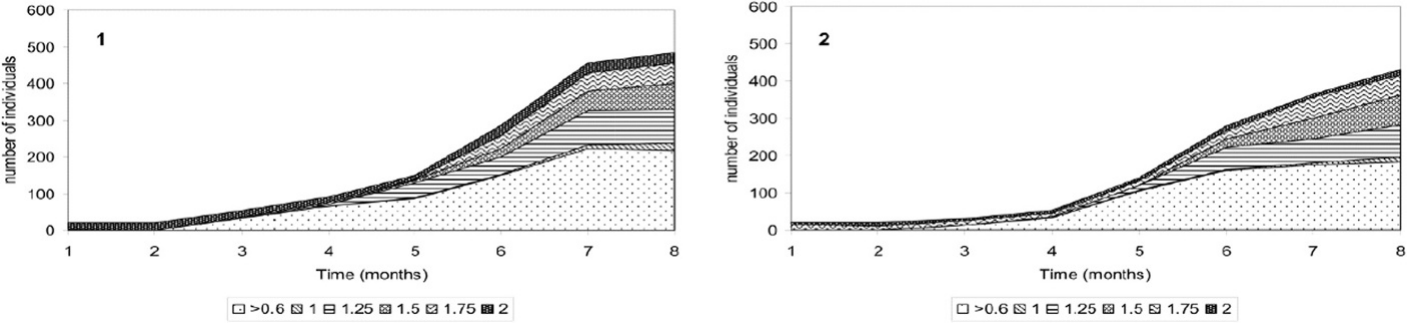
**Development of the populations of in the microcosms, according to the six size classes (in mm).** 1. Snails, sediment and water from meso-olgotrophic Lake I. 2. Snails, sediment and water from eutrophic Lake II.

Incubation of both populations derived from both the meso-oligotrophic and the eutrophic lake in microcosms supplemented with detritus and excess of lettuce showed that the reproductive output and population development in both lake environments was potentially similar in terms of size and reproductive output, i.e. number of individuals (Figure 5). During the first phase of the experiment (first two months), growth from the initially present 1.75 mm snails into 2.00 mm snails was observed. During the second phase (3 months onwards), reproduction (appearance of 0.60 mm snails) and growth to larger size classes were successful. When the numbers of 0.60 mm snails leveled off (at month 7), the numbers of adult snails still increased. The representation of 1.00 mm snails was very low, indicating a fast growth into the 1.25 mm size class. After eight months the snail densities were approximately 8,000 and 7,900 individuals/m2 in microcosm 1 and 2, respectively.

## Discussion

A regular development of the studied size classes within the *P. antipodarum* population in the eutrophic lake was observed. In contrast, in the meso-oligotrophic lake, the larger size classes contributed much less to the population despite a clear seasonal pattern visible in the number of developmental stages and nearly-neonates in the brood pouch. Clonal differentiation within populations of *P. antipodarum* is a well-known determinant of population dynamics. In New Zealand, diploid sexual individuals and asexual triploid clonal individuals of *P. antipodarum* (Wallace^1992^) coexist (e.g. Dybdahl and Lively^1995^; Jokela et al.^2003^), in which different subpopulations may differ in their life-history traits (e.g. Morley^2008^). Throughout Europe, however, males and sexual populations seem to be very rare and genotypic diversity is extremely low (Wallace^1985^; Hauser et al.^1992^; Jokela et al.^2003^; Gaino et al.^2008^). The populations investigated in this study only consisted of females and clones unlikely differed in genotype, especially since the lakes are located close to each other (<1 km) and they are interconnected. Moreover, passive transport of *P. antipodarum* by adherence to fish and waterfowl has been observed (Gaino et al.^2008^ and references therein; Miura et al.^2011^) and in both lakes, gulls (*Larus ridibundus*) are frequent visitors. Major differences between the two lakes are trophic state, food quantity and quality, which may thus have contributed considerably to the observed variation in population dynamics. This mechanism seems supported by our control microcosm experiment. In laboratory experiments (Dorgelo^1991^), shell growth rates of *P. antipodarum* (0.60–1.50 mm snails) were higher under conditions reflecting the eutrophic lake (lake sediment, detritus and water) than under conditions that reflected the meso-oligotrophic lake, whereas egg size and duration of the embryonic development, as well as shell growth rates in neonates proved to be unaffected, irrespective of their lake of origin. The development of *P. antipodarum* populations in the meso-oligotrophic lake thus revealed that meso-oligotrophic conditions resulted in reduced growth and distorted size-distributions and development of the population over time.

Trade-offs are considered important in life-history theory, yet they are difficult to detect in natural environments (Stearns^1989^). Population responses to environmental stress, food limitation or poor quality of the available food often implicate trade-offs between competing physiological demands, such as growth and reproduction, but energy is often allocated to growth at the cost of reproduction to increase chances of survival in relatively larger organisms. Despite the clear adverse effect of meso-oligotrophic conditions on growth in our study, the *per capita* production of nearly-neonates consistently did not differ between both populations over the survey period. This suggests that *P. antipodarum* populations consistently allocated energy toward reproduction rather than growth under meso-oligotrophic conditions, thereby contrasting our expectations. Various life-history strategies have been observed for other invertebrate species. For instance, under food depletion, two aquatic snail species (*Stagnicola elodes* and *Physella gyrina*) showed inverse strategies of internal resource investment. One species invested in growth, whereas the other species invested in reproduction (Rollo and Hawryluk^1988^). Likewise, zebra mussels (*Dreissena polymorpha*) in the same Maarsseveen lakes (Dorgelo^1993^) and sea urchins (Thompson^1982^) have also been observed to allocate energy in reproduction rather than growth under food-limiting conditions, suggesting that investments in reproduction at the expense of growth may be a more widespread strategy in aquatic detrital food webs.

A recent study incubated populations of *P. antipodarum* collected from the field in treatments with contrasting food abundances, and observed clear negative, density-dependent effects of food limitation on reproductive output (Neiman et al.^2013^). The experimental set up used in the present study does not allow any firm conclusions on whether the *P. antipodarum* populations in the meso-oligotrophic lake were in fact food limited, but it could be speculated that meso-oligotrophic conditions coincides with reduced food availability. In that case, it might be possible that effects of food limitation on life-history strategies at shorter time periods may differ from long-term effects of food limitation potentially experienced under meso-oligotrophic conditions. It is also possible that a continuous absence of food items may more severely affect reproduction as compared to natural systems that are overall food-limited, but periodically receive excess food. Following a similar reasoning, it was recently posed that peaks in food abundance may be an important driver for life-history strategy upon stress and food limitation or variability (e.g. algal blooms) in food abundance (Kooijman^2013^). The proposed ‘waste-to-hurry hypothesis’ suggests that small species (e.g. copepods and cladocerans) waste resources for the purpose of remaining small, growing fast, and responding rapidly with population numbers to temporal variations in primary production (e.g. algal blooms). It is possible that this concept extends to larger invertebrates in aquatic detrital food webs, especially considering the apparent responses of a variety of aquatic detritivores, including *P. antipodarum* in this study, and mussels, snails and isopods observed previously (Rollo and Hawryluk^1988^; Dorgelo^1993^; Lau et al.^2013^) to seasonal changes in food availability. Detrital food webs are fueled in large part by periodic algal blooming events and riparian litter loss (Goedkoop and Johnson^1996^), in which a large part (>50%) becomes trapped in sub-surface sediments (Herbst^1980^; Metzler and Smock^1990^). It seems feasible that peaks in food abundance may also be a potentially important driver for invertebrate macro-faunal life-history strategy in detrital food webs.

In conclusion, long-term surveys of natural *P. antipodarum* populations under both meso-oligotrophic and eutrophic conditions revealed that snails in the meso-oligotrophic lake showed reduced growth and a smaller size compared to snails in the eutrophic lake, while the numbers of eggs and nearly-neonates per adult snail did not differ significantly between the two populations.

## Authors’ original submitted files for images

Below are the links to the authors’ original submitted files for images. Authors’ original file for figure 1 Authors’ original file for figure 2 Authors’ original file for figure 3 Authors’ original file for figure 4 Authors’ original file for figure 5
