# Supplementary figures and images for: Dynamics of natural populations of the dertitivorous mudsnail Potamopyrgus antipodarum (Gray) (Hydrobiidae) in two interconnected Lakes differing in trophic state
Source: Springerplus. 2014 Dec 15;3:736. doi: 10.1186/2193-1801-3-736 (PMC4320230; doi:10.1186/2193-1801-3-736)

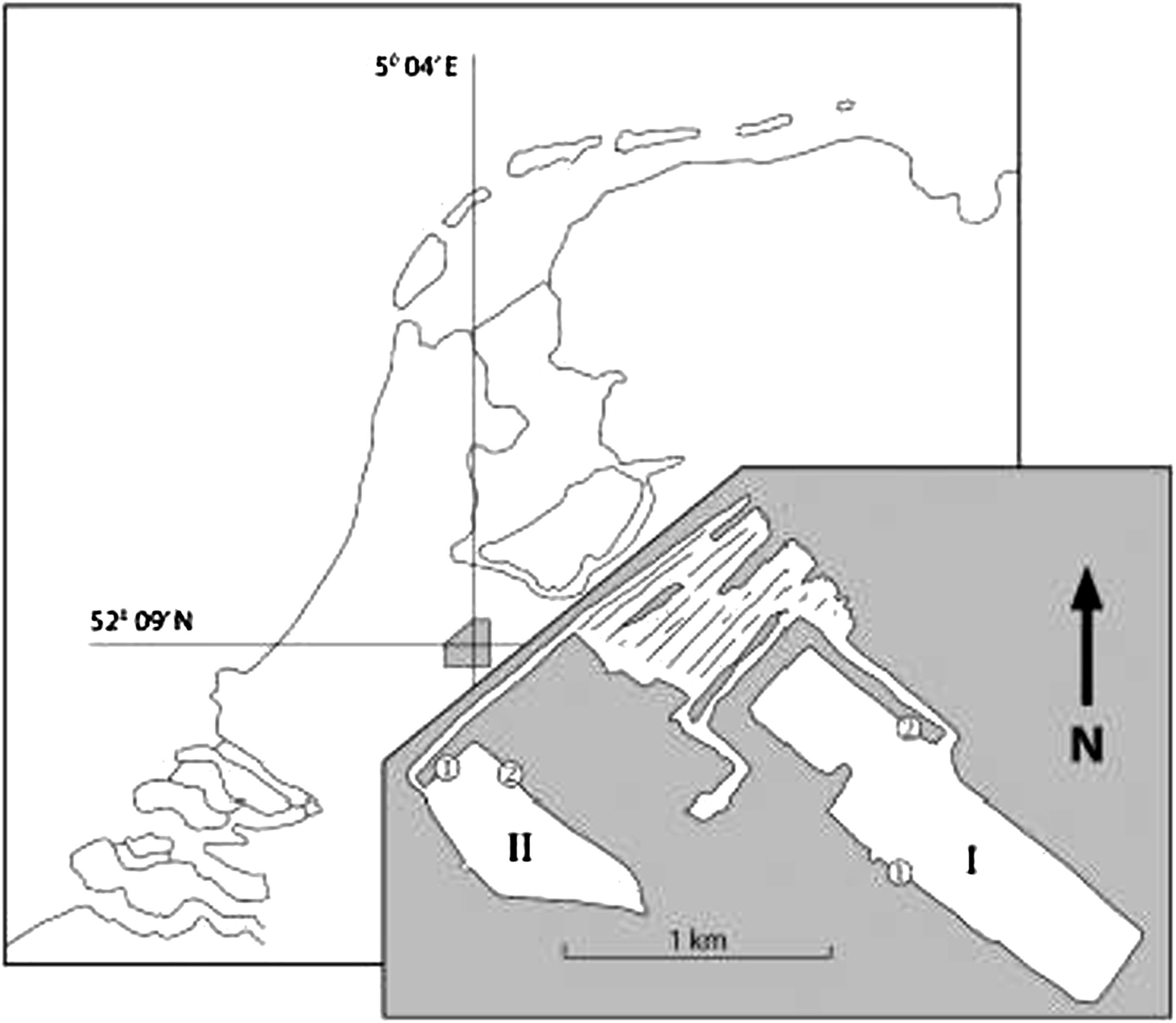

Supplement: Supplementary file 1 — Authors’ original file for figure 1 [file 40064_2014_1508_MOESM1_ESM.tiff]

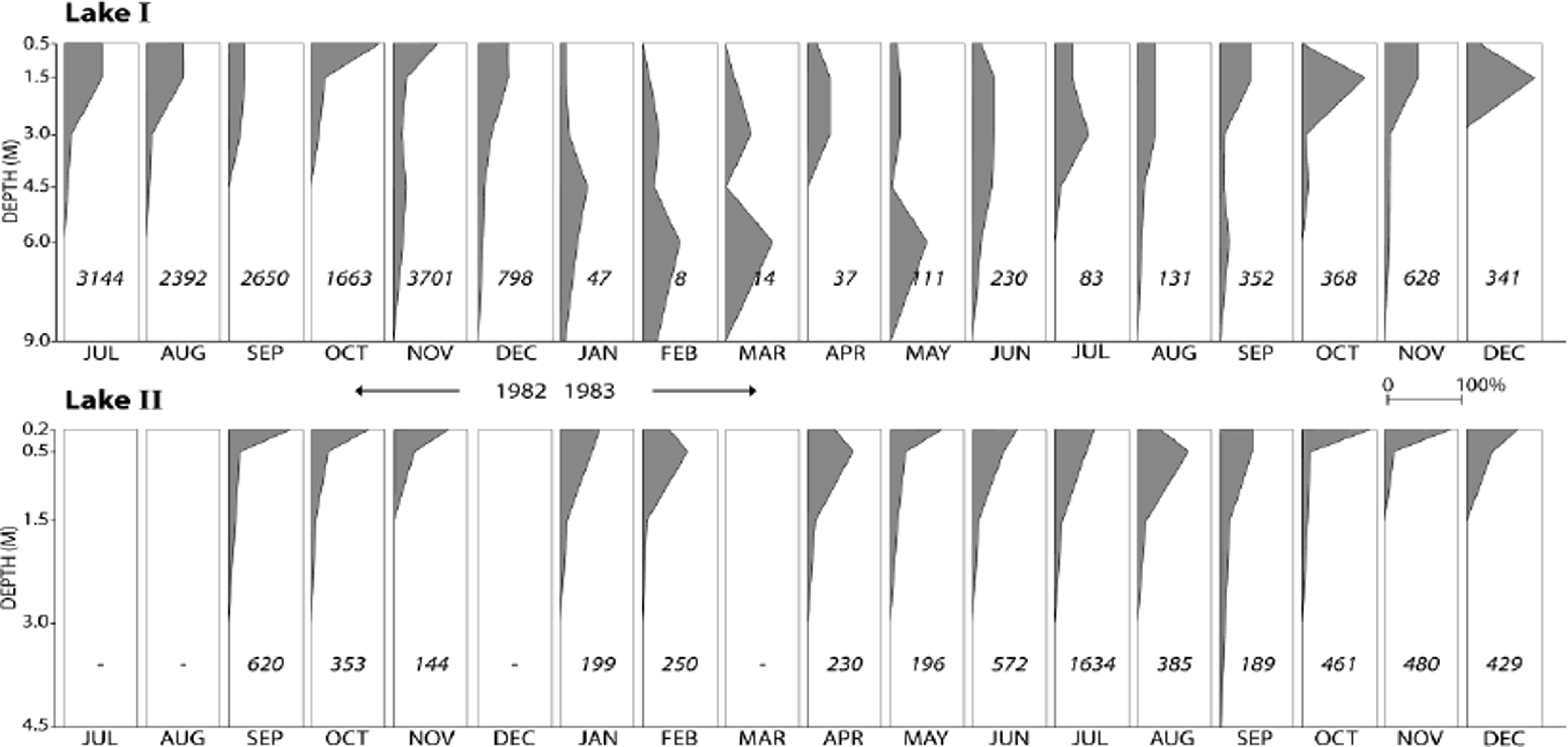

Supplement: Supplementary file 2 — Authors’ original file for figure 2 [file 40064_2014_1508_MOESM2_ESM.tiff]

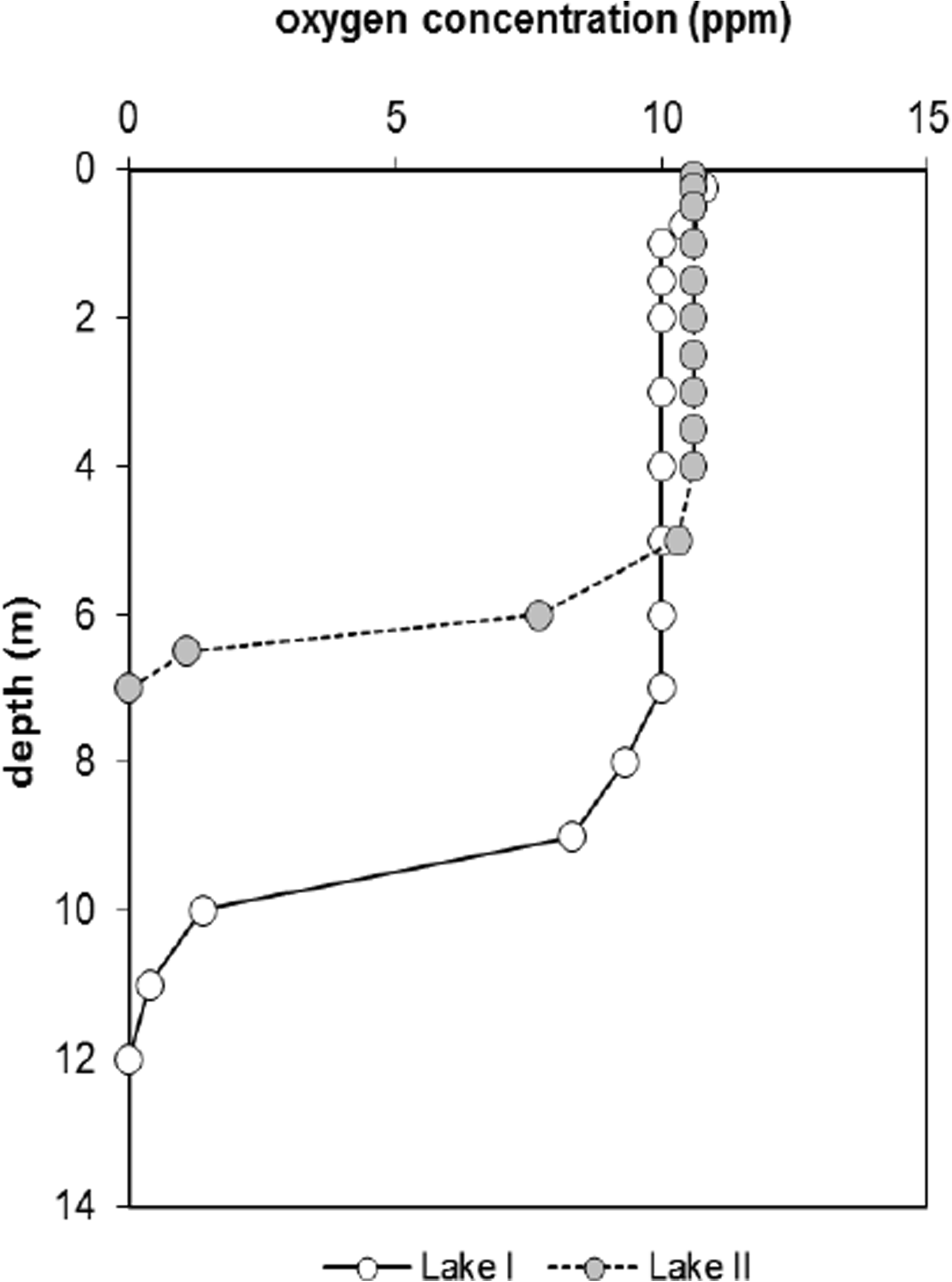

Supplement: Supplementary file 3 — Authors’ original file for figure 3 [file 40064_2014_1508_MOESM3_ESM.tiff]

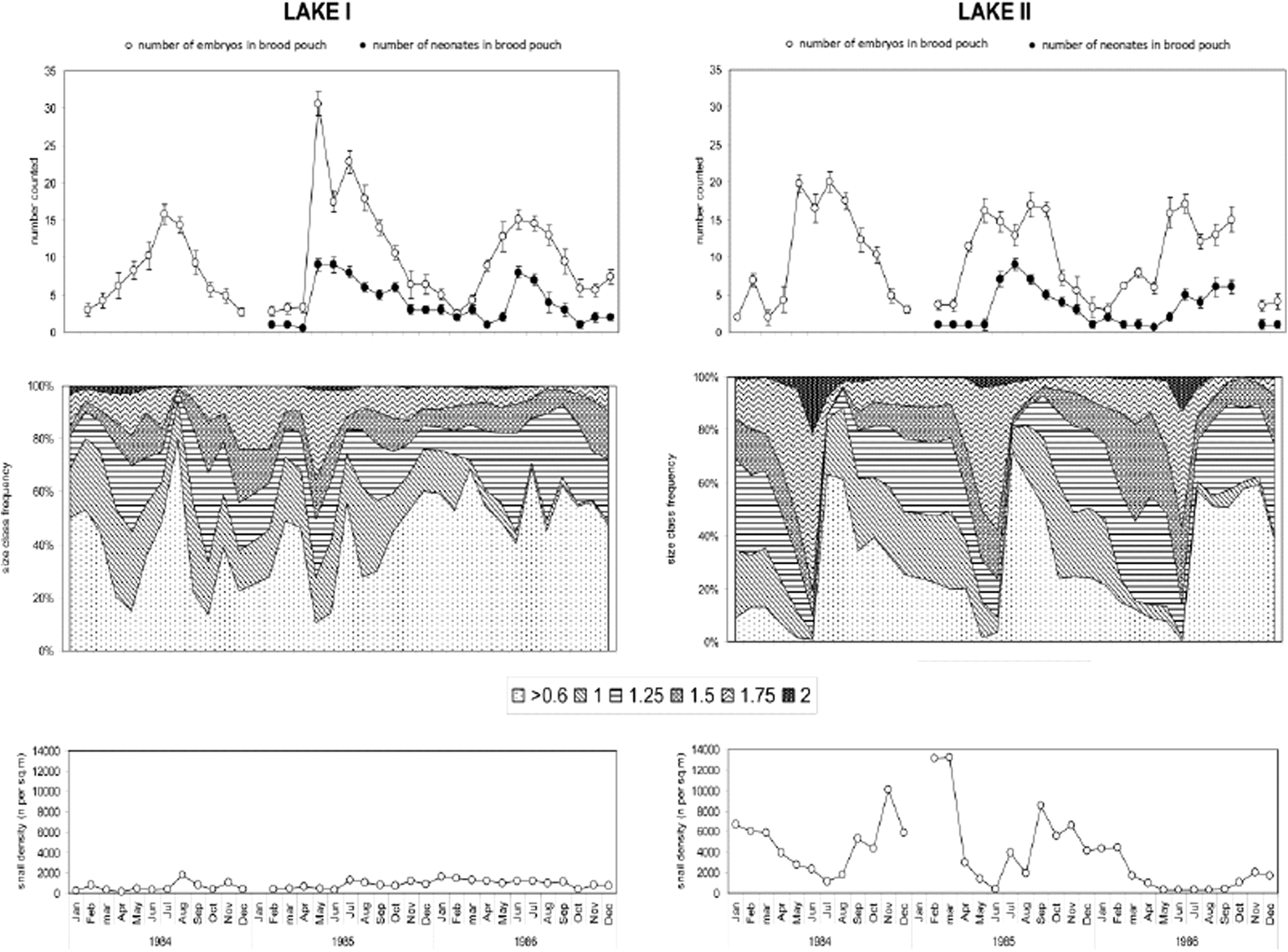

Supplement: Supplementary file 4 — Authors’ original file for figure 4 [file 40064_2014_1508_MOESM4_ESM.tiff]

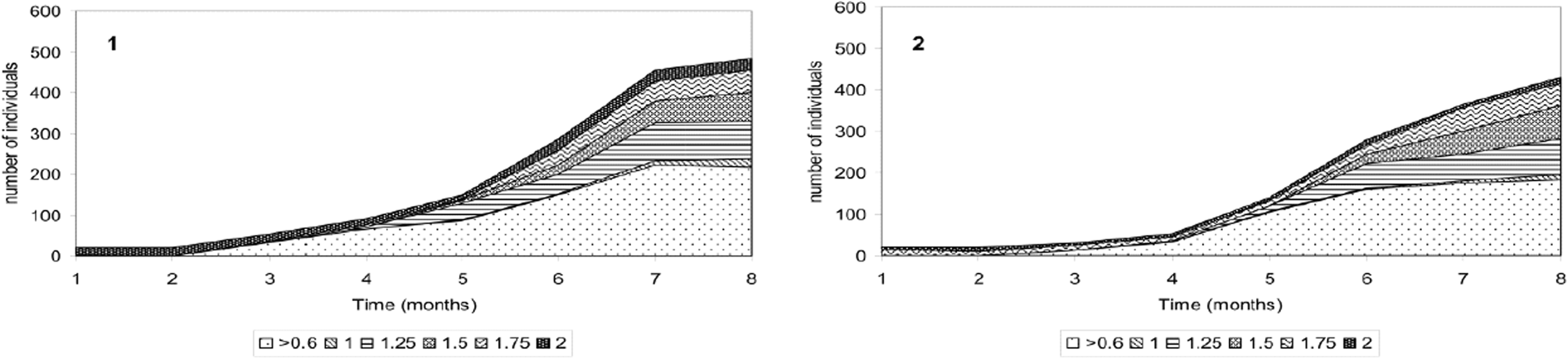

Supplement: Supplementary file 5 — Authors’ original file for figure 5 [file 40064_2014_1508_MOESM5_ESM.tiff]
